# Prevalence of mental distress and associated factors among medical students of University of Gondar, Northwest Ethiopia: a cross-sectional study

**DOI:** 10.1186/s12888-022-04174-w

**Published:** 2022-08-02

**Authors:** Gidey Rtbey, Shegaye Shumet, Belete Birhan, Endalamaw Salelew

**Affiliations:** 1 Department of Psychiatry, College of Medicine and Health Sciences, University of Gondar, Gondar, Ethiopia; 2 College of Medicine and Health Sciences, Wolayita Sodo University, Wolayita Sodo, Ethiopia

**Keywords:** Mental distress, Medical students, University of Gondar

## Abstract

**Background:**

Mental distress is the most common problem among medical students. This is associated with severe consequences of lack of empathy for their patients, committing medical errors, and suicidal ideations and attempts. However, there is limited data on this aspect where the study was conducted especially in this segment of the population. Considering its seriousness, this study will have pivotal input information to plan possible interventions for the future. So, this study is aimed at assessing the prevalence of mental distress and its associated factors among medical students of the University of Gondar, Northwest Ethiopia, 2021.

**Methods:**

An institutional-based cross-sectional study was conducted using a stratified random sampling technique to get a total of 438 study subjects from April 15–30/2021. Mental distress data were collected using a self-administrated questionnaire of the 10-item Kessler Psychological Distress Scale. Data was entered to Epi-data version 4.6.02 and cleaned, coded, and analyzed using STATA version 14.

**Results:**

The prevalence of mental distress among medical students was 193(45.95%) with 95% CI (41.2, 50.7). In multi-variable logistic regression being female sex (AOR = 4.5, 95% CI = 2.66, 8.12), lack of interest towards field of study (AOR = 4.4, 95%, CI = (2.18, 8.78), current alcohol use (AOR = 5.8, 95% CI = 3.03, 11.15), monthly pocket money < 735 Ethiopian birr (AOR = 3.1, 95% CI = 1.53, 6.04), extremely high test anxiety (AOR = 3.9, 95% CI = 1.27, 11.88), family history mental illness (AOR = 2.5 95% CI = 1.12, 5.53) and poor social support (AOR = 4.2, 95% CI = (1.94, 9.16) were significantly associated with mental distress.

**Conclusion and recommendation:**

Prevalence of mental distress among medical students of University of Gondar was found to be higher when compared to previous studies among this population in Ethiopia. It is recommended that the school of medicine should give undue attention to address those identified factors by establishing counseling centers to minimize mental distress.

## Introduction

Mental distress is defined as the unique discomforting, emotional state experienced by an individual in response to a specific stressor or demand that results in harm, either temporary or permanent, to the person [1]. This unpleasant mental or emotional state could be manifested with anxiety, depressive and somatic symptoms such as sleep problems, headache, and backache [2–5]. Mental distress can also be presented with confusing emotions, hallucinations, and related symptoms without actually being ill in a medical sense [6].

Reviews of different studies on mental distress among medical students found a high prevalence of stress, depression, and anxiety, with levels consistently higher than in the general population [7]. Medical education is aimed at producing competent physicians who are capable of taking the responsibility for advancing public health issues and achieving high levels of patient-centered care [8]. To equip this capability medical students are expected to go through many years of stressful studying and persistent clinical training [9]. Students’ ongoing struggle to become highly qualified healthcare providers might affect their mental and emotional health unintentionally [10].

Worldwide medical students are at risk of mental distress and reduced satisfaction in life when compared to other populations [11]. Previous studies have shown high rates of depression, anxiety, and stress among medical students throughout the world [11, 12]. A systematic review carried out on 40 published studies among US and Canadian medical students revealed that mental distress was significantly higher than the general population [13]. Another systematic review was done on 16 identified studies outside North America in the English-speaking world; the prevalence of mental distress among medical students ranged between 12.2 and 96.7% [14].

A study of Egyptian medical students’ showed a high Positive Symptom Distress Index (PSDI) of 30.1% [15]. A systematic study done in Nigeria showed that mental distress was present in 25.2% of medical students [16], and in Ethiopia prevalence of mental distress among medical students ranged between 30 and 35.2% [17–19].

The potential consequences of mental distress among medical students include decrements in academic performance and clinical practice, stress-induced disorders, and deteriorating performance [20]. Furthermore, it can also be associated with severe consequences like lack of empathy for their patients and colleagues and doing medical errors, chronic stress, burn-out syndrome, depression, and, in extreme situations, suicidal ideations, and attempts [21–23]. A prospective cohort study conducted in the United States indicated that residents with depression and burnout out; which could be direct effects of mental distress commit higher medical errors than their counterparts [24].

According to different literature reviewed possible factors that contribute for developing mental distress were; dealing with stressors specific to professional training such as the new information flow and input overload, examinations, chances of failure, lack of leisure time, workload, relationships with peers, and career choices [15]. Moreover, being female sex, coming from a rural background, having a history of substance use, high academic demands, financial problems, and apprehension about the future [11, 18].

Despite mental health services being included in the national health policy of Ethiopia, interventions against the problems are very limited and the lack of information about the problem is a contributory factor for poor mental health care services [25].

Caring and trying to understand the sufferings of health care providers is the most crucial thing to enhance the quality of health care service. So this study conducted will help to provide an input information about the prevalence and associated factors of mental distress among medical students of the University of Gondar for future interventions. The previous studies don’t show exactly the current mental distress status of medical students in which many things are changed due to the covid-19 pandemic impacts on the teaching–learning process and the northern Ethiopian conflict might had its own share for mental distress where this study was conducted. Furthermore, this study included predictors like test anxiety and reason to join a medical school which were not incorporated in the previous studies.

## Methods and materials

### Study design, setting, and period

An institutional-based cross-sectional study was conducted at the University of Gondar, College of Medicine and Health Sciences from April 15–30/2021 which is found in the historical city of Gondar, Amhara region. It is located 737 km far from Addis Ababa, the capital city of Ethiopia. The town has an altitude and longitude of 12˚36ˈN 37˚28ˈE with an elevation of 2,133 m above sea level.

College of Medicine and Health Sciences is one of the five campuses of the University of Gondar in which the study was conducted. The college was founded in 1954 as Public Health College. It has four schools: a school of medicine, a school of pharmacy, a school of biomedical science, a school of nursing, and one institute: the institute of public health. According to the University of Gondar College of Medicine and Health sciences registrar office report students who registered to this college in the academic year of 2020/2021 were 3023 and out of these 1472 were medical students. However, during the data collections period only 1177 medical students were academically active.

### Participants

The study was conducted among University of Gondar medical students. Medical students who were academically active and present on the campus during the data collection period were included in the study whereas medical students who were severely ill and in difficulties to respond the questionnaires were excluded.

### Sample size and Sampling procedure

The sample size was determined by using a single population proportion by taking the prevalence of mental distress 35.2%; a study reported from Jimma University, Ethiopia [18] with; a 4% margin of error, 95% confidence, and assuming 10% nonresponse rate the final sample size was taken 438.

Medical students of University of Gondar were stratified based on their phase of study as pre medicine (first year), preclinical (second and third year), clinical (fourth and fifth year), and internship (sixth or last year) but since there were no intern students during data collection we only took the first three strata. As data obtained from the University of Gondar registrar’s office indicated that the total number of medical students during data collection was 1177(premedicinne = 105, preclinical = 435, and clinical = 637). Then proportional allocation of study subjects for each strata was calculated and 39, 162, and 237 medical students were drawn from premedicine, preclinical and clinical respectively. Finally, computer generated lottery method was used to select study participants from each given strata.

### Study variables

#### Dependent variable

Mental distress.

#### Independent variables

Sociodemographic factors such as age, sex, religion, marital status, phase of study, living background and monthly pocket money. Psychosocial factors included were social support, having close friends, reason to join medical school, interest towards field of study, religious practice, failures in exam and test anxiety. Substance and behavioral factors such as alcohol, khat, smoking and other substances (cannabis, shisha, cocaine, and pethidine).

### Data collection tool and procedure

Data was collected using an English version of a self-administered questionnaire having six parts. The first part included the sociodemographic characteristics of participants. The second part assesses test anxiety of medical students using the Westside Test Anxiety Inventory(WTAI); A self-reported questionnaire of 10 statements with each response is scored with a 5-point likert scale [1–5] yielding a total test anxiety score ranging from 10 to 50 points. WTAI scale combines six items assessing impairment and four items on worry and dread related to exams. “30–34” moderately high test anxiety, 35–39 high test anxiety, and 40–50 extremely high test anxiety [26].

Even though WTAI is not validated in Ethiopia, the tool was used to assess test anxiety among Addis Ababa University medical students with internal consistency (Cronbach’s alpha) of 0.94 [27].

The third part of the questionnaire was Kessler Psychological Distress Scale (K10) which was used to assess mental distress; the outcome variable with a cut-off point ≥ 20 and with a sensitivity of 84.2% and specificity of 77.8% [28, 29]. The K10 is a widely used tool to assess mental distress in the preceding month [30].

Both the 10-item and 6-item versions (K10 and K6) were validated in Ethiopia, with the 10-item version showing superior validity with internal consistency (Cronbach's alpha) of 0.90 [29]. Each item is rated from 1 to 5, from “none at all” to “all the time”. The total score for the 10-item scale is 50, ranging from 10 to 50. Respondents were asked about experiencing symptoms of mental distress over the past 1 month. In the current study, a participant who scored 20 and above was considered as having mental distress [30]. In this study the internal consistency (cronbach’s alpha) of mental distress was found to be 0.89.

The next three parts were social support, substance use behaviors, and other psychosocial and clinical related factors respectively. Social support of the students was assessed using the Oslo-3 social support scale which ranges from 3 to 14, those respondents who scored “3–8” were considered as having poor social support, score “9–11” moderate social support, and scored “12–14” as having strong social support [31].

In addition to the above different substance use behavior like alcohol, chat, smoking, and other substance uses were assessed using ASSIST (by asking questions about current use; for non-medical purpose with in the last 3 months) [32]. Clinical factors like chronic medical illness and current mental illness were also assessed by using semi-structured questions.

Data were collected with three BSc psychiatry professionals and the responsibility of the data collectors was to provide the questionnaires after obtaining their voluntariness from study subjects. The principal investigator was checking the filled questionnaire for completeness, and solve forwarded problems timely during data collection. During data collection all the data collectors and participants were using covid-19 prevention protocols like face mask, sanitizer, washing hands and social distancing.

### Data quality control

To assure the data quality, training was given to data collectors and supervisors preceding the data collection time. Before the actual data was collected, the questionnaire was tested on 5% of the total sample size (22 individuals) among Bahirdar University medical students. The collected data were checked for completeness before the actual data entry and incomplete data were discarded.

### Data processing and analysis

Data was entered to Epi-data version 4.6.00 and cleaned, coded, and analyzed using STATA version 14. Then, the data was analyzed to generate descriptive statistics: means, frequency, percentages and standard deviations, using STATA. Binary logistic regression was used to analyze the data.

Firstly every variable was checked using bi-variable logistic regression to get variables which had association with dependent variable and then variables which had *p*-value of less than 0.25 were entered into multi-variable logistic regressions for further analysis. Adjusted odds ratio with 95% CI was computed for variables having a *p*-value less than 0.05 in multi-variable logistic regression model and considered as significantly associated with the dependent variable.

## Results

### Sociodemographic characteristics of study participants

A total of 420 students fully responded to the questionnaires yielding a 96% response rate. 18 of the questionnaires were incompletely filled. Male respondents accounted 254(60.5%). The mean age of respondents was 22.86 years (SD ± 1.85). The majority of the respondents 360(85.7%) were single and 297(70.7%) were Orthodox Christianity followers. About 227(54.1%) of the study participants were in the clinical phase of study followed by the preclinical phase 156(37.1%) and 335(79.8%) of them were from an urban background (Table 1).

**Table 1:** Distribution of Sociodemographic characteristics of medical students' of the University of Gondar, 2021 (*n* = 420)

Variables	Categories	Frequency (*n* = 420)	Percentages
Sex	Male	254	60.5
	Female	166	39.5
Age	< 24	344	81.9
	> 24	76	18.1
Marital status	Single	360	85.7
	Married/in relationship	60	14.3
Religion	Orthodox	297	70.7
	Islamic	47	11.2
	Protestant	64	15.2
	Others	12	2.9
Phase of study	Premedicine	37	8.8
	Preclinical	156	37.1
	Clinical	227	54.1
Living background	Rural	85	20.2
	Urban	335	79.8
Monthly pocket money (In Ethiopian birr)	< 735	90	21.4
	735–1176	91	21.7
	> 1176	239	56.9

NB. Others = Adventist, Pagan, Atheist, and Jehovah’s Witness

1 US dollar = 52 Ethiopian birr

### Psychosocial, clinical, and substance related characteristics of study participants

Related to the psychosocial characteristics of respondents about 216(51.4%) had moderate social support and 247(58.8%) of the study participants sometimes participated in religious practices. The majority 299(71.2%) of respondents joined medical school by their personal preferences and 324(77.1%) participants had an interest towards their field of study. About 57(13.6%) and 25(5.9%) of respondents had a family history of mental illness and known chronic medical illness respectively. Regarding substance use of the students, about 102(24.3%) of them were current alcohol users (Table 2).

**Table 2:** Distribution of psychosocial, clinical and substance related behaviors of medical students' of the University of Gondar, 2021 (*n* = 420)

Variables	Categories	Frequency(420)	Percentage
Social support	Poor	97	23.1
	Moderate	216	51.4
	Strong	107	25.5
Test anxiety	No test anxiety	298	70.9
	Moderate high test anxiety	66	15.7
	High test anxiety	22	5.2
	Extremely high test anxiety	34	8.1
Religious practice	Always	142	33.8
	Sometimes	247	58.8
	Not at all	31	7.4
Having close friends	Yes	382	90.9
	No	38	9.1
Frequent conflict with friends	Yes	30	7.1
	No	390	92.9
Reason to join medical school	Personal preferences	299	71.2
	Family influence	90	21.4
	Others^a^	31	7.4
Interest toward field of study	Yes	324	77.1
	No	96	22.9
Ever had failed an exam	Yes	116	27.6
	No	304	72.4
Family history of mental illness	Yes	57	13.6
	No	363	86.4
Past history of mental illness	Yes	17	4.1
	No	403	95.9
Known chronic medical illness	Yes	25	5.9
	No	395	94.1
Current alcohol use	Yes	102	24.3
	No	318	75.7
Current smoking	Yes	16	3.8
	No	404	96.2
Current khat use	Yes	12	2.9
	No	408	97.1
Others^b^	Yes	17	4.2
	No	403	95.9

Others^a^ = Employment, Societal preferences, Misinformation

Others^b^ = Cannabis, Shisha, Cocaine and Pethidine

### Prevalence of mental distress among medical students of University of Gondar

**Fig. 1:**
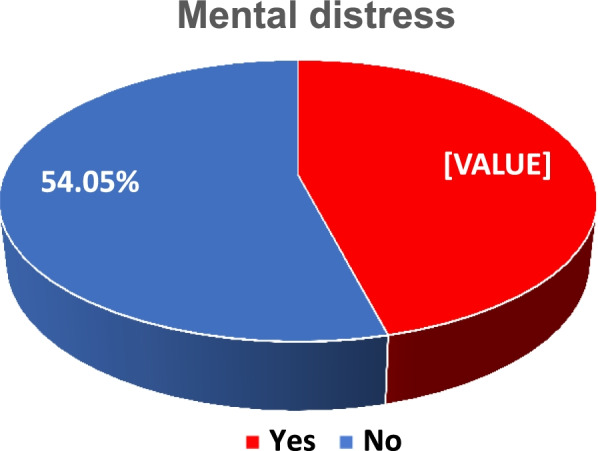
Prevalence of mental distress among medical students of University of Gondar, 2021 (*n* = 420)

In this study, the overall prevalence of mental distress among medical students was 193(45.95%) with 95% CI (41.2, 50.7) (Fig. 1).

### Factors associated with mental distress among medical students

In bi-variable logistic regression sex, phase of the study, monthly pocket money, social support, test anxiety, family history of mental illness, history of mental illness, known chronic medical illness, ever had failed in the exam, the reason to join a medical school, interest towards the fiel of study, frequent conflict with friends, having close friends, religious practice, current alcohol use, and cigarette smoking were associated (*p*-value < 0.25) with mental distress among medical students.

In multivariable analysis being female sex, low monthly pocket money, poor social support, extremely high test anxiety, family history of mental illness, lack of interest in field the of study and current alcohol user were associated (*p*-value < 0.05) with mental distress.

The odds of developing mental distress among females were 4.5 times higher when compared to males(AOR = 4.5, 95% CI = 2.66, 8.and 12), the odds of developing mental distress among those who had monthly pocket money less than 735 Ethiopian birr were 3.1 times higher than those who had > 1176 Ethiopian Birr (AOR = 3.1, 95%CI = 1.53, 6.04). The odds of having mental distress among those who had lack of interest towards their field of study were 4.4 times higher than their counterparts (AOR = 4.4, 95% CI = 2.18, 8.78), and the odds of developing mental distress among respondents who were alcohols user were 5.8 times higher than those who didn’t use alcohol (AOR = 5.8, 95%CI = 3.03, 11.15) (Table 3).

**Table 3:** Bi-variable and multivariable analysis of factors associated with mental distress among medical students' of the University of Gondar, 2021 (*n* = 420)

Variables	Categories	Mental distress		95% CI	
		Yes	No	COR	AOR
Sex	Male	93	161	1.00	1.00
	Female	100	66	2.62( 1.75, 3.92)	4.5(2.66,8.12)**
Phase of study	Premedicine	22	15	2.07(1.02, 4.21)	2.17(0.84, 5.60)
	Preclinical	77	79	1.37(0.91, 2.07)	0.80(0.46, 1.41)
	Clinical	94	133	1.00	1.00
Monthly pocket money	< 735	61	29	3.48(2.08, 5.82)	3.1(1.53, 6.04)*
	735–1175	42	49	1.41(0.87, 2.31)	1.3(0.68, 2.45)
	> 1176	90	149	1.00	1.00
Social support	Strong	33	74	1.00	1.00
	Moderate	85	131	1.45(0.88, 2.38)	0.93(.51, 1.72)
	Poor	75	22	7.64(4.08,14.32)	4.2(1.94, 9.16)**
Test anxiety	No test anxiety	121	177	1.00	1.00
	Moderately high	31	35	1.29(0.758, 2.21)	1.72(0.88, 3.36)
	High test anxiety Extremely	13	9	2.11(0.87, 5.09)	2.9(0.97, 8.54)
	high anxiety	28	6	6.83(2.74, 16.98)	3.9(1.27, 11.88)*
Known Chronic medical illness	Yes	17	8	2.64(1.11, 6.27)	1.38(0.46, 4.11)
	No	176	219	1.00	1.00
Family history of mental illness	Yes	43	14	4.36(2.30, 8.25)	2.5(1.12, 5.53)*
	No	150	213	1.00	1.00
Ever had failed an exam	Yes	69	44	2.31(1.48, 3.59)	1.76(.97, 3.18)
	No	124	183	1.00	1.00
Interest towards field of study	Yes	120	204	1.00	1.00
	No	73	23	5.39(3.20, 9.07)	4.4(2.18, 8.78)**
Reason to join medical school	Personal preference	118	181	1.00	1.00
	Family influence	52	38	2.1(1.30, 3.38)	1.19(0.62, 2.32)
	Other	23	8	4.4( 1.90, 10.18)	1.28(0.46, 3.59)
Having close friends	Yes	162	220	1.00	1.00
	No	31	7	6.01(2.58, 13.99)	1.75(0.58, 5.24)
Frequent conflict with friends	Yes	20	10	2.5(1.14, 5.49)	1.9(0.68, 5.31)
	No	173	217	1.00	1.00
Religious practice	Always	52	90	1.00	1.00
	Sometime	119	128	1.6(1.05,2.45)	1(0.59, 1.82)
	Not at all	22	9	4.23(1.81, 9.87)	1.6(0.55, 4.69)
Current alcohol use	Yes	70	32	3.46(2.15, 5.57)	5.8(3.03,11.15)**
	No	123	195	1.00	1.00
Current smoking	Yes	16	7	2.84(1.14, 7.05)	0.32(0.08, 1.20)
	No	177	220	1.00	1.00

^*^Significant association (*p*-value < 0.05), **Significant association (*p*-value < 0.01)

Other = Employment, societal preferences and misinformation

## Discussion

Medical school is known to be the area of pressure and overwhelming distress. The current study revealed that the prevalence of mental distress was found to be 45.95% with a 95% CI (41.2, 50.7). This result is high as compared to studies done in Hawassa (30%) [17], Jimma (35.2%) [18], and Addis Ababa (32.6%) [19], Ethiopia. This inconsistency might be due to assessment tools difference; in which the previous studies used SRQ20 and the setting in which participants of the current study could be repeatedly exposed to severely wounded soldiers as a result of the neighbor’s devastating war. In addition to this, the reason why this study is higher might be students are more distressed nowadays as a result of the covid-19 pandemic impacts on the educational issues [33]. It was also higher than the studies done in Bangalore (30.39%) [20] and Amritsar (34.4%)[2] of India, south Karnataka (32.2%) [34], Saudi Arabia (35.8%) [35]. This discrepancy might be occurred due to environmental factors, infrastructure accessibility and assessment tools used such as SRQ20 and GHQ whereas the current study used Kessler psychological distress scale (K10).

This study was lower when compared to studies conducted Hong Kong, China (87%) [36], Bangladesh (54%) [37], and Pandit Bhagwat Dayal University of India (53.3%) [38]. This discrepancy could be due to differences in tools used and other sociocultural differences. Furthermore, the study setting might be contributed to this variation in which Hong Kong is known to be a stressful city [36]. However, this result goes in line with studies done in Brazil (44.7%) [39], Dutch medical students (48%) [40] and study done in Malaysia (41.9%) [41].

Regarding to the factors associated with mental distress, current study revealed that being female was significantly associated with mental distress. Studies from Hong Kong, China [36], South Karnataka[34] and Amritsar [2] of India and Gondar [42] and Jimma [18], Ethiopia support this finding. The possible reasons for the higher prevalence of mental distress among female students could be the affective nature of their response to stressors, domestic violence, learned helplessness and hormonal changes during menstruation [43].

This study also indicated that having low monthly pocket money was associated with mental distress. Studies similar to this finding include Jimma, Ethiopia [18]. This might be due to those who had low monthly pocket money could face different financial stresses like the inability to afford material which are necessary for academic purposes and financial difficulties to engage in social issues like get togethers [44].

Having poor social support was one of the significant predictors of mental distress in the current study. Students who had poor social support from significant others were more than four times higher in developing mental distress. This was supported by studies from Hawassa [5] and Gondar [42], Ethiopia. This could be possibly due to the effect of getting insufficient emotional, instrumental and informational support from significant other. This could lead students to cope poorly in stressful situations and decrease their resilience, in which indirectly make them more vulnerable for developing mental distress.

Moreover, the odds of developing mental distress among those who had extremely high test anxiety were around four times higher than those participants who didn’t have test anxiety. This finding was similar to a study conducted on Malaysian medical students [45, 46]. The possible reason for this might be poor academic performance and fear of failure could lead students to feel incapable and have low self-esteem which could lead them to develop mental distress.

Students who had a family history of mental illness in this study were significantly associated with mental distress which was similar to studies from Jimma and Gondar [18, 42], Ethiopia. This could be explained by genetic predisposition and living conditions within the families. In addition, caring for a mentally ill family member may also be an additional stress that contributes to a higher prevalence of mental distress [47].

The current study revealed that students who had a lack of interest towards their field of study were more than four times higher to experience mental distress than their counterparts. This finding is similar with study done in Gondar [42], Ethiopia. This could be reasoned out by studying a field without interest prone students to distress and dissatisfaction.

Furthermore, this study indicated that respondents with current alcohol use were found to be higher to experience mental distress than those who didn’t. This finding was similar to a research study done by Jimma [18] and Hawassa [5] of Ethiopia. This could be because, alcohol use leads to inefficiency in functioning, impaired relationship, and sleep deprivation. Moreover, it is also associated with increased absenteeism from class and poor academic performance which can further lead to mental distress [48]. However, since the study was cross-sectional, it is difficult to ascertain the direction of causality.

### Limitation of the study

This study had some important limitations that should be kept in mind. Cross sectional nature of the study design made it difficult to establish a cause-effect relationship. Since data were collected using self-administered questionnaires there might be reporting bias and some questions also assess history which is prone to recall bias. In addition to this, since intern students were not part of this study this might create result variations.

## Conclusion and recommendation

The overall prevalence of mental distress among medical students of University of Gondar was found to be higher when compared to previous studies done on mental distress among other Ethiopian medical students. Being female sex, lack of interest towards field of study, current alcohol use, extremely high test anxiety, low monthly pocket money, poor social support, and family history of mental illness were significantly associated with mental distress. So considering this, it is recommended that it would be better if school of medicine establish counselling centers for early detection and take corrective measures to decrease the burdens of this problem. In addition to this, gender office of University of Gondar should create conducive environment to female medical students for helping one another.

## Data Availability

The data that support the findings of this study is available but some restrictions may apply to the availability of these data as there are some sensitive issues. However, data is available from the corresponding authors upon reasonable request.

## Abbreviations

AOR: Adjusted Odds Ratio
ASSIST: Alcohol, Smoking and Substance Involvement Screening Test
CI: Confidence Interval
COR: Crude Odds Ratio
GHQ: General Health Questionnaires
K10: 10-Item Kessler Psychological Distress Scale
SRQ20: 20-Item Self-Reporting Questionnaire
US: United States
WTAI: West side Test Anxiety Inventory
